# Complete mitochondrial genome of *Leucopsarion petersii* (Gobiiformes, Gobiidae) assembled from next-generation sequencing data

**DOI:** 10.1080/23802359.2025.2559716

**Published:** 2025-09-16

**Authors:** Jeong-Soo Gim, Dong-Hyun Hong, Jeong-An Gim, Maurice Lineman, Gea-Jae Joo, Hyunbin Jo

**Affiliations:** ^a^ Department of Pet Health Care, Busan Health University, Busan, Republic of Korea; ^b^ Department of Integrated Biological Science, Pusan National University, Busan, Republic of Korea; ^c^ Department of Medical Science, Soonchunhyang University, Asan, Korea; ^d^ RCF Experimental School, Beijing, People’s Republic of China

**Keywords:** Complete mitochondrial genome, *Leucopsarion petersii*, gobiiformes

## Abstract

We report the complete mitochondrial genome of *Leucopsarion petersii* from specimens collected in Geoje City, South Korea. Using next-generation sequencing, we determined the mitochondrial genome is a circular DNA molecule of 16,525 bp, comprising 13 protein-coding genes, 22 tRNA genes, and two rRNA genes. Maximum likelihood phylogenetic analysis using 12 mitochondrial genomes showed this species clustered with other Gobiiformes, providing first insights into phylogenetic relationships within this order.

## Introduction

*Leucopsarion petersii* Hilgendorf, 1880, is an anadromous fish widely distributed in shallow coastal areas throughout the Japanese Archipelago and Korean Peninsula (Matsui 1986; Kim and Park 2002). In South Korea, this species is restricted to a very narrow range in the downstream reaches of the Sanyang-chun stream, which flows into the southern part of the East Sea (Sea of Japan). Historical and current data indicates a limited or very weak population structure in the South Sea of Korea (Kim et al. 2022).

Population decline has been observed in these basins due to river system modifications and construction activities (DeBoer et al. 2019; Jo et al. 2019). Preservation of genetic resources from native species is crucial for protecting declining populations (Vrijenhoek 1998; Lakra et al. 2007; Alexander Kenechukwu et al. 2019). While several genetic markers of *L. petersii* have been previously studied, including the cyt b gene, nuclear myh6 gene (Kokita and Nohara 2011), and control region sequences, a complete mitochondrial genome analysis has not yet been conducted for this species.

In this study, we sequenced the complete mitochondrial genome of *L. petersii* collected from Geoje-do, South Korea. This genomic data will enhance the genetic information available for *L. petersii* and provide essential support for species identification, evolutionary studies, phylogenetic analysis, and biogeographical research within the genus *Leucopsarion*.

## Materials and methods

**Figure 1.**
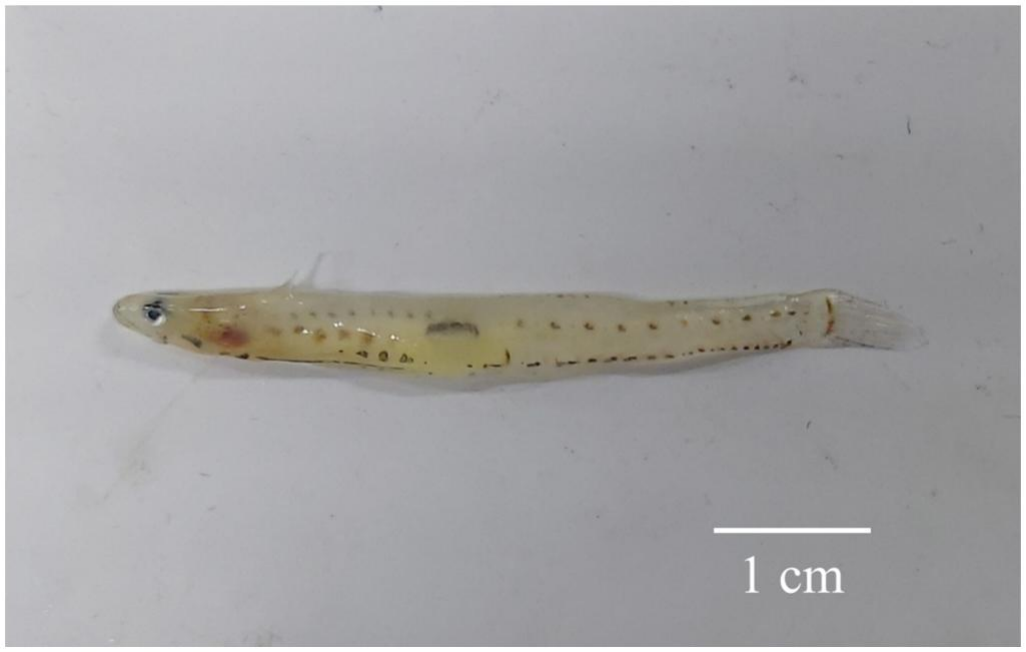
*Leucopsarion petersii* collected from the Sanyang-chun stream in Geoje City, South Korea. Main diagnostic characters include a thin, elongated, eel-like body. It lacks scales but possesses a swim bladder and a small pelvic fin. The photo was taken by Jeong-Soo gim at the Specimen Museum of Pusan National University in december 2023, and there are no copyright issues.

The samples of *L. petersii* were collected on 2 December 2023 from Sanyang-chun stream, Geoje City, South Korea (34°49′46.06″N, 128°35′45.49″E). The specimens were fixed in 99.9% ethanol and were stored in the Specimen Museum of Pusan National University (https://limnology.pusan.ac.kr/limnology/index.do) under the voucher number PNUBIO-0110010102 (contact Dong-Hyun Hong, hdh1201@pusan.ac.kr). Based on morphological characteristics, the collection was identified by Kim and Park (2002). *L. petersii* (total length: 51 mm; weight: 0.2 g) are presented in Figure 1. Specific permission is not needed as no endangered or protected species were involved. *L. petersii* specimens were homogenized using a PrecellysV R 24D homogenizer (Bertin, Montigny-le-Bretonneux, France), and DNA was extracted with a DNeasy Blood & Tissue Kit (Qiagen, Hilden, Germany). Library preparation (Illumina TruSeq DNA PCR-free; Cat. No. 20015963), genome assembly (SPAdes, V 3.12), and data preparation for DNA sequencing (101 bp paired-end reads on an Illumina NovaSeq 6000) were conducted by Macrogen Inc. (Seoul, Korea). The assembled genome was analyzed using MEGA-X software (Kumar et al. 2016). Phylogenetic analysis was performed with IQ-TREE software (ver. 1.6.12; Nguyen et al. 2015) using the protein-coding gene sequences and maximum likelihood method. To root the phylogenetic tree and better understand evolutionary relationships within Gobiiformes, *Perca fluviatilis* (Perciformes: Percidae) was included as an outgroup taxon based on its close phylogenetic relationship to Gobiiformes. The annotated mitochondrial genome sequence of *L. petersii* has been deposited in the National Center for Biotechnology Information database under the accession number PP727280.

## Results

### Mitochondrial genome features

**Figure 2.**
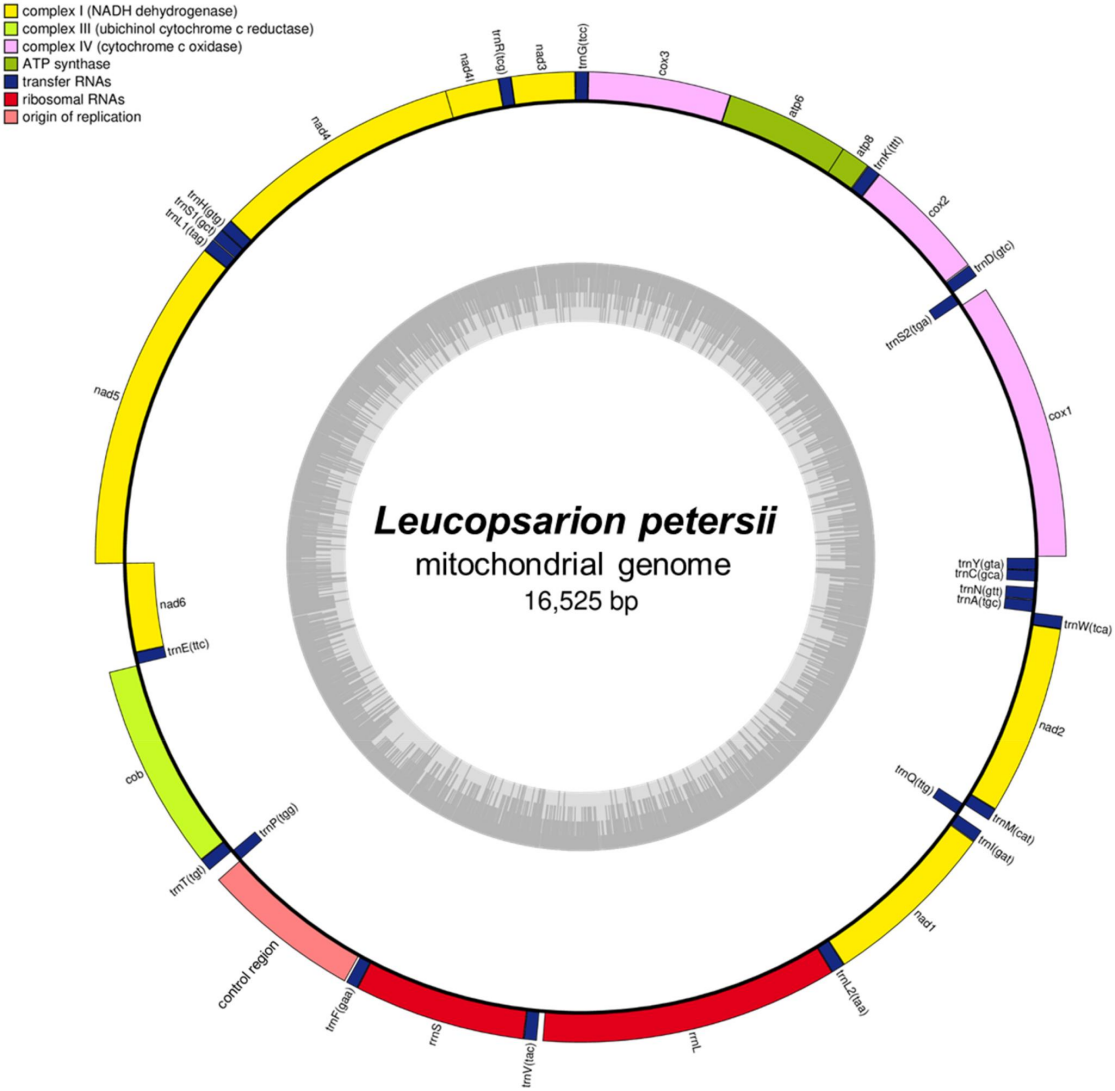
Complete mitochondrial genome pattern maps of *leucopsarion petersii* from the Sanyang-chun stream in Geoje City, South Korea. Genes shown outside and inside the outer circle are transcribed in counterclockwise and clockwise directions, respectively. The inner circles represent the genome scale, GC content and distributions of short tandem repeats, long tandem repeats and the dispersed repeats, respectively. The colored parabola in the center circle represents the dispersed repeats.

**Figure 3.**
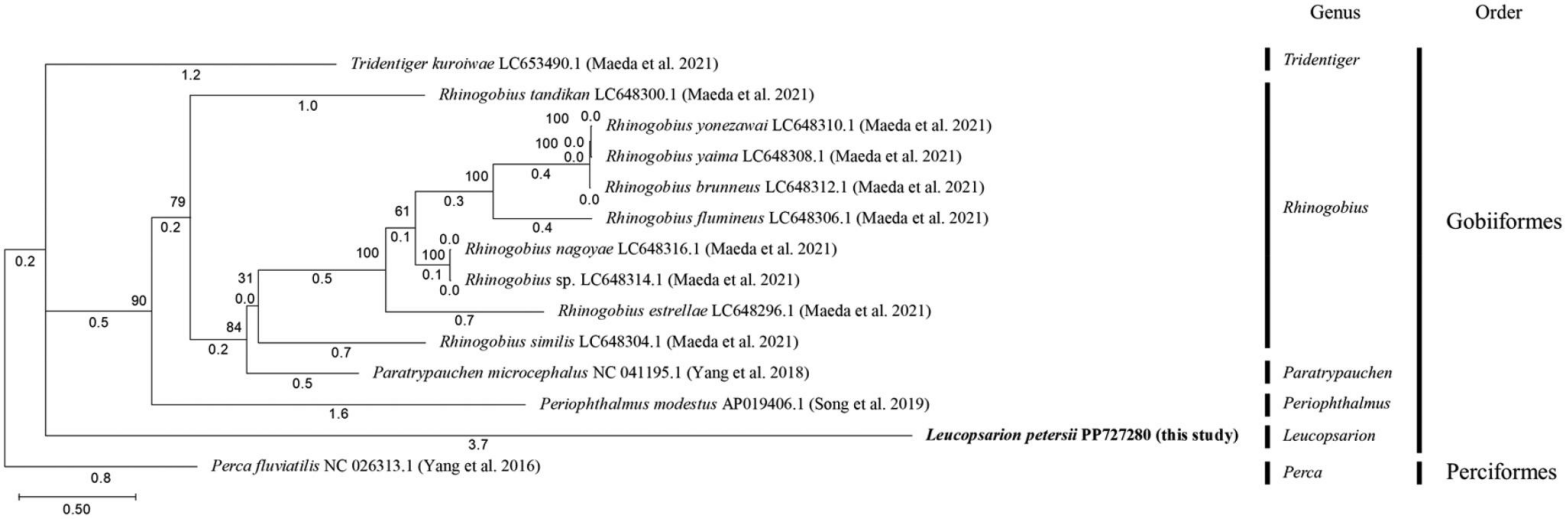
Maximum likelihood trees inferred from coding sequences of all 12 mitochondrial genes of Gobiiformes. The heuristic search (using the 50% majority-rule with 1000 bootstrap replicates) shows *Leucopsarion petersii* as a related species to the family Gobiiformes, with a relationship similar to that of other phylogenies. The GenBank accession numbers of the species are given after the scientific names, and are as follows: *Leucopsarion petersii* (PP727280; This study), *Paratrypauchen microcephalus* (NC_041195.1; Yang et al. 2018), *Periophthalmus modestus* (AP019406.1; Song et al. 2019), *Rhinogobius brunneus* (LC648312.1; Maeda et al. 2021), *R. estrellae* (LC648296.1; Maeda et al. 2021), *R. flumineus* (LC648306.1; Maeda et al. 2021), *R. nagoyae* (LC648316.1; Maeda et al. 2021), *R. similis* (LC648304.1; Maeda et al. 2021), *Rhinogobius* sp. (LC648314.1; Maeda et al. 2021), *R. tandikan* (LC648300.1; Maeda et al. 2021), *R. yaima* (LC648308.1; Maeda et al. 2021), *R. yonezawai* (LC648310.1; Maeda et al. 2021), *Tridentiger kuroiwae* (LC653490.1; Maeda et al. 2021), *Perca fluviatilis* (NC_026313.1; Yang et al. 2016).

The circular mitochondrial DNA (mtDNA) of *L. petersii* is 16,525 bp in length and includes 13 protein-coding genes (atp6, atp8, nad1, nad2, nad3, nad4, nad4L, nad5, nad6, cox1, cox2, cox3, and cob), two ribosomal RNA genes (rrnL and rrnS), 22 transfer RNA genes (trnA, trnC, trnD, trnE, trnF, trnG, trnH, trnI, trnK, trnL1, trnL2, trnM, trnN, trnP, trnQ, trnR, trnS1, trnS2, trnT, trnV, trnW, and trnY), and the control region (Figure 2). The genome’s A + T base composition is 54.23%, with the genes having an A + T content of 53.82%. The G + C base composition of the genome is 45.77%, and the genes’ G + C content is 46.18%. Phylogenetic analysis, conducted using IQ-TREE software, with *Perca fluviatilis* as an outgroup, revealed that *L. petersii* clusters with other species within the Gobiiformes order, confirming its taxonomic placement within this group. This analysis was based on the mitochondrial genome of *L. petersii* and comparable mtDNA sequences from Gobiiformes obtained from GenBank following a BLASTN search (Figure 3).

## Discussion and conclusion

This study presents the first complete mitochondrial genome characterization of *L. petersii* collected from South Korea. The mitogenome of *L. petersii* shows typical characteristics in terms of gene number and arrangements, while displaying distinct features in its nucleotide composition. The mitochondrial genome length variation is primarily attributed to differences in nucleotide composition regions, with this species exhibiting an extended A + T-rich region compared to reference species (*Rhinogobius* sp.; LC648314.1; Maeda et al. 2021), while simultaneously showing a shorter G + C-rich region. This pattern of nucleotide composition contributes to the overall genomic structural variation observed in *L. petersii*.

Phylogenetic analysis revealed that *L. petersii* is most closely related to *Tridentiger kuroiwae* (Mukai et al. 1996) (Figure 3). The inclusion of *Perca fluviatilis* as an outgroup successfully polarized the phylogenetic tree, providing clear evolutionary directionality and confirming the monophyletic nature of the Gobiiformes species analyzed in this study. While the complete mitogenome of Japanese *L. petersii* has not been previously reported and was thus excluded from the current phylogenetic tree, future studies incorporating this data would enhance our understanding of intraspecific variation. The genomic data presented here provides a foundation for understanding the phylogenetic position, evolutionary relationships, and biogeographical patterns of *L. petersii* within the Gobiiformes family.

## Supplementary Material

250212_mtDNA_appendix_re.docx

## Data Availability

The mitochondrial genome data are available with the accession number of PP727280 in the GenBank of NCBI (https://www.ncbi.nlm.nih.gov/). The associated BioProject, SRA, and Bio-Sample numbers are PRJNA1137556, SRR29892735 and SAMN42621800, respectively.
