# Supplementary material for: Complete mitochondrial genome of Leucopsarion petersii (Gobiiformes, Gobiidae) assembled from next-generation sequencing data
Source: Mitochondrial DNA B Resour. 2025 Sep 16;10(10):972–5. doi: 10.1080/23802359.2025.2559716 (PMC12444920; doi:10.1080/23802359.2025.2559716)

**Supplementary Material**

Complete mitochondrial genome of *Leucopsarion petersii* (Gobiiformes, Gobiidae) assembled from next-generation sequencing data

Jeong-Soo Gim^a^, Dong-Hyun Hong^b^, Jeong-An Gim^c^, Maurice Lineman^d^, Gea-Jae Joo^b^ and Hyunbin Jo^a*^*+*

^a^ Department of Pet Health Care, Busan Health University, 46241, Busan, Republic of Korea.

^b^ Department of Integrated Biological Science, Pusan National University, 46241 Busan, Republic of Korea

^c^ Department of Medical Science, Soonchunhyang University, Asan, 31538, Korea

^d^ RCF Experimental School, Chaoyang District, Beijing, People’s Republic of China.

^*^Corresponding Author: Hyunbin Jo, Prof. PhD, Department of Pet Health Care, Busan Health University, 46241, Busan, Republic of Korea.

Tel.: +82-200-3236; Fax: +82-200-1599; E-mail: prozeva@bhu.ac.kr

**This PDF file includes:**

FIGURES S1

In mitochondrial DNA (mtDNA) sequence analysis, assessing sequencing depth and coverage is crucial to ensure high assembly quality of organelle genomes. Errors in genome assembly can lead to inaccurate downstream analysis, making depth and coverage analysis essential (Yang et al., 2023). Therefore, we analyzed the mtDNA of *Leucopsarion petersii* to generate a sequencing depth and coverage map (Figure S1).

**References**

Ni, Y., Li, J., Zhang, C., & Liu, C. 2023. Generating sequencing depth and coverage map for organelle genomes. doi: dx.doi.org/10.17504/protocols.io.4r3l27jkxg1y/v1

Figure S1. Generating sequencing depth and coverage map for mitochondrial genome of *Leucopsarion petersii*.


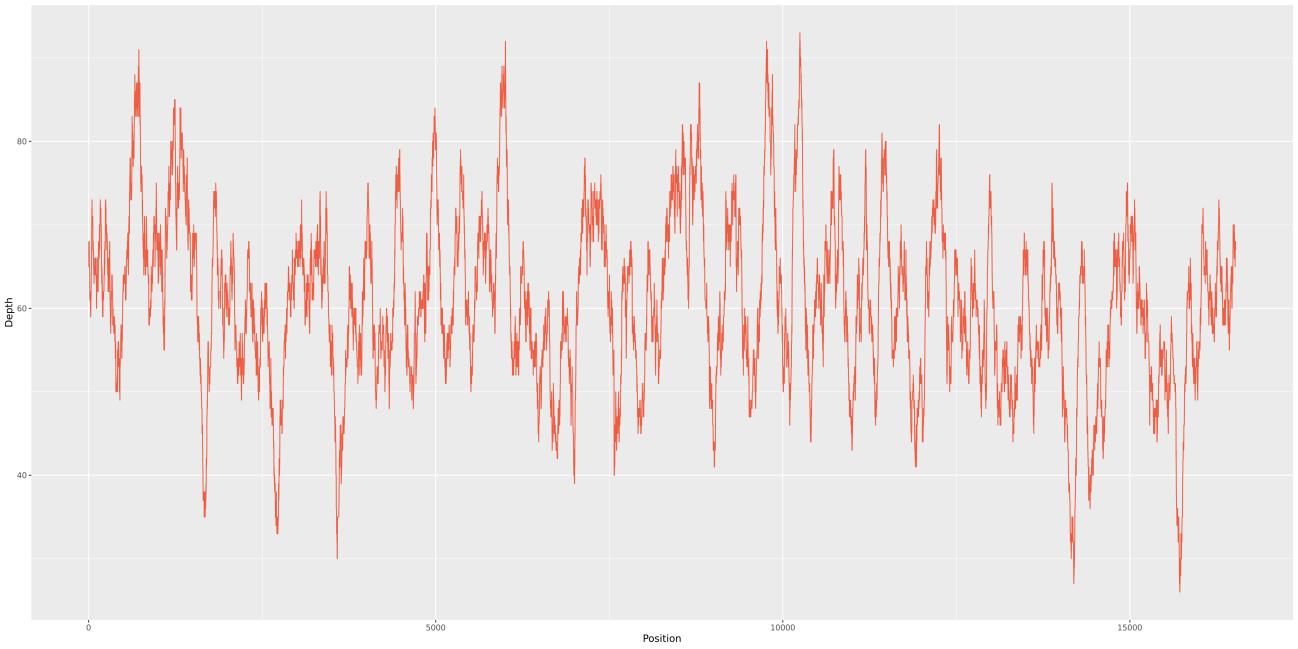

Supplement: 250212_mtDNA_appendix_re.docx [file TMDN_A_2559716_SM3427.docx]
